# Ultrasound-guided large-core needle biopsies of breast lesions: analysis of 962 cases to determine the number of samples for reliable tumour classification

**DOI:** 10.1038/sj.bjc.6602303

**Published:** 2004-12-21

**Authors:** G Sauer, H Deissler, K Strunz, G Helms, E Remmel, K Koretz, R Terinde, R Kreienberg

**Affiliations:** 1 Department of Obstetrics and Gynecology, University of Ulm Medical School, Prittwitzstr. 43, Ulm 89075, Germany; 2 Department of Pathology, University of Ulm Medical School, Ulm, Germany

**Keywords:** breast cancer, large-core needle biopsy, ultrasound

## Abstract

The objective of this one-institutional study was to determine the number of large-core needle biopsies (LCNB), under three-dimensional ultrasound (3D-US) validation, that are sufficient to obtain a reliable histological diagnosis of a sonographically detectable breast lesion. Over an 28-month period, 962 sonographically guided LCNB were performed under 3D-US validation to assess 962 breast lesions. All biopsies were carried out with an automated core biopsy device fitted with 14-gauge (22 mm excursion) needles. Data of 962 biopsied breast lesions were gathered. Surgical follow-up was available for 659 lesions. Breast malignancies were diagnosed by ultrasound-guided LCNB with a sensitivity of 98.2% by performing three cores per lesion. In few cases, the open surgical specimen revealed the presence of invasive carcinomas in contrast to initial LNCB-based classification as ductal carcinomas *in situ* (DCIS, 11 lesions), lobular carcinoma *in situ* (one lesion), and atypical ductal hyperpasia (one lesion). Owing to disagreement between classification based on breast-imaging and histological findings, eight of these tumours were subsequently excised. Of the lesions that were removed at the patients’ requests despite benign LCNB diagnosis, two were infiltrating carcinoma and one a DCIS. We demonstrate that three 3D-US-guided percutaneous core specimens are sufficient to achieve tissue for a reliable histological assessment of sonographically detectable breast lesions and allow the detection of malignancies with high sensitivity and low rate of false-negative diagnoses.

The widespread mammography screening programmes for the early detection of breast cancer gave rise to a tremendous number of biopsies taken in order to determine the nature of sono- or mammographically diagnosed breast abnormalities. In this diagnostic concept, a percutaneous image-guided large-core needle biopsy (LCNB) has become an alternative to the open surgical biopsy to remove tissue for histological assessment (Smith DN *et al*, 2001; Smith WL *et al*, 2001; Crowe *et al*, 2003a; Pijnappel *et al*, 2004). Large-core needle biopsy can be performed either under mammographic or sonographic guidance. In general, the sonographic approach is preferred if the lesion can be visualised by both methods. Besides the comparison of different imaging tools, controversy has largely centred around reliability and reproducibility of this approach (Crowe *et al*, 2003b), the required amount of tissue, and the minimum number of samples which have to be evaluated for a reliable classification of breast lesions. Most authors suggest that five core specimens per lesion are required for exact tumour classification (Parker *et al*, 1994; Philpotts *et al*, 2003); however, this has not been universal practice in clinical routine. Several studies have addressed this question regarding stereotactic guidance and have determined five specimens to be required for a reliable tumour classification (Liberman *et al*, 1994; Brenner *et al*, 1996). To our knowledge, no comparable large-sized study for ultrasound (US)-guided biopsy has been performed.

In this investigation, we analysed data derived from US-guided biopsies of 962 breast lesions in 906 patients to evaluate reliability and reproducibility of a three-dimensional (3D) US-guided automated LCNB technique. We demonstrated that the number of core samples could be reduced by 3D-US-guided validated LCNB (ensuring correct placement of the biopsy needle) without decrease of diagnostic accuracy.

## MATERIALS AND METHODS

### Image-guided large-core needle biopsies of breast lesions

**Figure 1:**
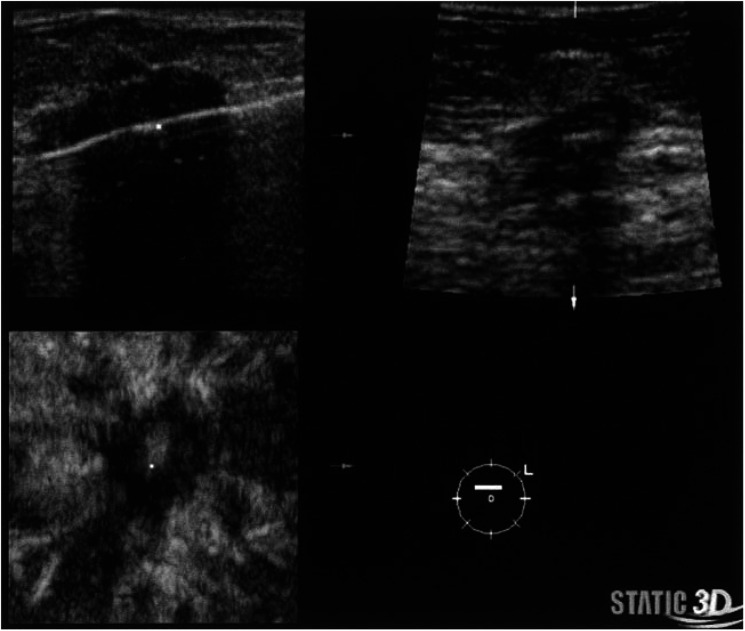
Multiplanar imaging based on a 3D-US volume data set during ultrasound-guided 14-gauge automated core biopsy proves that needle has transversed mass as central hit.

During a period of 28 months, 3D-US-guided biopsies were taken from 906 patients referred to our clinic with a total of 962 palpable or nonpalpable breast lesions that had been initially detected by palpability, mammography, and/or ultrasound imaging. No initial biopsies by surgical excision, stereotactic biopsy, or any other means were performed of those lesions before US-guided LCNB. We performed a two-dimensional (2D) ultrasound examination (Voluson 730, Kretztechnik through GE Medical Systems, Zipf, Austria; 3D-US transducer 5–13 MHz, 30° volume sector) with the patients in supine position and elevated arms to localise the primarily detected or additional lesions in the same or contralateral breast. A sonographic classification of all lesions into benign or malignant was performed according to characteristics that have been described previously (Stavros *et al*, 1995). Prior to the first breast biopsy, an informed consent was obtained and a history of blood coagulation problems requested from each patient. No laboratory tests were performed unless the patient was under anticoagulation therapy or reported a history of coagulopathy. All biopsies were performed by means of a core needle throw (22 mm excursion) automated biopsy gun (Bard-Magnum Biopsy Instrument, Covington, GA, USA) fitted with 14-gauge needles. In order to achieve uniformity of methods, positioning of the LCNB needle was performed exclusively by three persons who had undergone dedicated training in LCNB techniques. The procedure was carried out strictly according to a standard protocol described previously, except the number of core specimen per biopsy (Parker *et al*, 1993). After the needle had been placed at the edge of the lesion under 2D-US guidance (pre-firing position), the 22 mm core needle throw was executed. Then, a 3D-US volume data set (10 Mb) was acquired and converted into a multi-planar imaging display to define the precise post-firing position of the needle (Figure 1). If a biopsy was recognised to be marginal or out of the lesion, additional biopsies were taken until at least two biopsies were central hits.

Whenever patient's concerns persisted despite a core biopsy with benign histology, either LCNB was repeated or an open surgical biopsy was carried out. In all other cases with benign core biopsy results in accordance with US images, follow-up mammography or US examination at 3-month intervals was recommended in accordance with the results of previous studies (Parker *et al*, 1994; Philpotts *et al*, 2003).

### Classification of lesions and generated data sets

Six parameters were recorded for all of the lesions: size (calculated longest diameter), palpability, sonographic classification, number of specimen per lesion, histological findings at LCNB, and excision biopsy. Of the four defined histological categories, the first was assigned to malignant lesions (infiltrating ductal, medullary, tubular, lobular, mucinous, papillary or other invasive carcinomas, not defined were lymphomas or metastatic diseases). The second category included all grades of ductal carcinomas *in situ* (DCIS). Since the differentiation between low-, intermediate-, or high-grade DCIS on the basis of small LCNB samples is an awkward task, no differentiation of grading was carried out. Category 3 included the so-called high-risk lesions like lobular carcinoma *in situ* (LCIS) and atypical ductal hyperplasias (ADH). Category 4 included benign lesions: fibroadenomas, areas of fibrocystic changes, scar and fat tissues, papillomas, and other benign lesions. Core needle biopsies with uncertain histological finding due to an insufficient amount of tissue were assigned to a separate group, all of which underwent surgical excision.

Agreement between surgical specimen or clinical follow-up and histological findings based on LCNB was assessed. Disagreement between histological finding in the LCNB and sonographic classification of the lesion in breast imaging was considered an indication for a subsequent excision biopsy. As a consequence, lesions expected to be malignant according to sonographic criteria that have been previously described (Stavros *et al*, 1995) were removed completely by open surgery even if LCNB-based histological evaluation had suggested a benign abnormality. All patients for whom core biopsy revealed ADH, LCIS, or DCIS underwent subsequent open surgical biopsy.

To describe the degree of agreement, three categories were defined: (1) cases with complete agreement between surgical specimen or clinical follow-up and LCNB specimen, (2) cases with disagreement between surgical biopsy findings and examination of LCNB specimen; and (3) cases with partial agreement, including those in which ADH was diagnosed on the basis of LCNB but DCIS or invasive carcinoma (high risk lesions underestimates) concluded from the surgical biopsy, and DCIS that were later diagnosed to possess an invasive component (DCIS underestimates).

### Statistical analysis

Overall and parameter-specific diagnostic yields were assessed, and comparison between LCNB specimen and histological finding at excision biopsy or clinical follow-up was determined with McNemar's test. Sensitivity and specificity of the LCNB technique to yield a diagnosis were calculated. For patients with benign LCNB results that did not undergo excision, follow-up imaging or cross-reference with other hospitals and cancer databases was performed to determine whether malignant lesions were subsequently diagnosed.

## RESULTS

**Figure 2:**
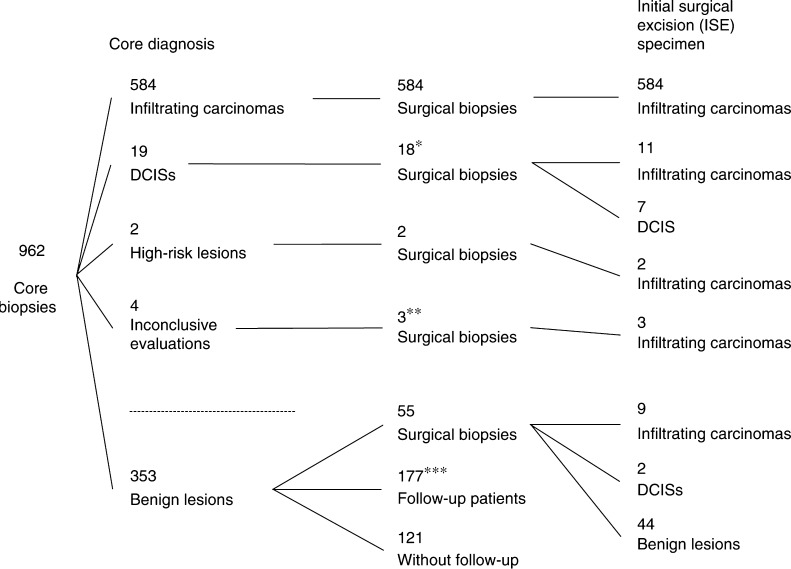
Follow-up outcome of all breast lesions sampled by LCNB (*n*=962). ^*^One patient refused excision biopsy despite LCNB-based DCIS diagnosis. ^**^One patient refused excision biopsy despite inconclusive LNCB diagnosis. ^***^Three of these patients underwent subsequent surgery which confirmed benign lesions, all others were without any changes.

Of the 962 lesions, 603 (63%) were malignant, two (0.2%) high-risk, 353 (37%) benign, and four (0.4%) insufficient for diagnosis at LCNB specimen. Of the 603 malignant specimens, 584 were infiltrating carcinoma and 19 DCIS; of the high-risk lesions, one was a LCIS, the other an ADH. In 659 cases, surgical follow-up by means of an excision biopsy was performed where the lesion was totally removed. Surgical excision yielded 615 malignancies (609 infiltrating carcinoma, seven DCIS) and 44 benign lesions (Figure 2). Of the 609 infiltrating carcinoma, 11 have been assigned to be DCIS (DCIS underestimates) and two high-risk lesions (high-risk underestimates) at LCNB specimen. Three cases also came out to be invasive, where LCNB did not yield sufficient material for diagnosis. Overall histological agreement between the LCNB specimen and finding at excision biopsy was highly significant (*P*=0.001). Two patients refused excision biopsy, where LCNB specimen showed DCIS or insufficient tissue for reliable tumour classification. The observed high cancer rate in this series reflects the fact that only lesions were biopsied where breast imaging yielded a lesion of BI-RADS III or higher. The average size was 2.5 cm (median 2.2 cm; range 0.2–11 cm). The sizes between benign and malignant lesions were statistically not different. In all, 236 lesions were palpable, 726 were not, without a difference between benign and malignant.

For the diagnostic assessment of 962 breast lesions, 2.044 3D-US-guided core specimens were obtained in a period of 28 months. In most (730, 75.9%) cases, two biopsies per lesion were sufficient to obtain a clear and reliable histological result. Only one core was taken in 31 (3.2%), three in 165 (17.2%), four in 12 (1.2%), and five in two (0.2%) of the sonographically detectable lesions. In few (22, 2.3%) cases, the number of biopsies was not available. The sensitivity of LCNB under 3D-US guidance to identify infiltrating breast lesions was 96.1% (24/616), the specificity 100%. The average of follow-up was 22.2 months (median 21; range 8–36 months). However, not following our recommendation to come in 3-month intervals for follow-up examinations, not all patients had returned for re-evaluation despite their remaining risk of misdiagnosed lesions. In all, 34% (121/353) patients with benign lesions have not returned for follow-up. For these patients, cross-reference with other institutions was performed, and no case of breast cancer was found. However, since cancer databases might not be 100% reliable, those 121 cases were excluded from statistical analysis. This did not alter the results. Three patients who underwent re-examination in our hospital for follow-up showed an increasing lesion size and subsequently underwent excision biopsy that yielded a benign diagnosis.

Among the 962 analysed lesions, almost all (603 of 616; 97.9%) malignancies were identified by LCNB and immediately surgically excised as a consequence. Of the 55 lesions that have been classified by LCNB to be benign, 29 lesions were determined to be BI-RADS IV and V lesions by breast imaging. Therefore, excision biopsy was carried out and yielded eight malignancies (seven infiltrating carcinoma, one DCIS) and 21 benign lesions. Of 23 breast tumours where LCNB and breast imaging yielded a benign diagnosis, and which have been removed, three came out to be malignant in the surgical specimen (two infiltrating carcinoma, one DCIS).

Analysis of data from surgical follow-up, performed in 659 of the lesions, revealed complete agreement between histological findings in LCNB and subsequent open surgical biopsies for 635 (96.2%) lesions. Partial agreement was found in 13 (2%) and clear disagreement only in 11 (1.8%) of the 659 excised lesions (Figure 2). Those 11 malignant lesions that were initially misdiagnosed by LCNB consisted of nine infiltrating carcinoma and two DCIS (Table 1).

All 11 LCNB misses occurred in lesions that were sonographically classified as masses and central hits of the needle. Eight of these lesions appeared as BI-RADS IV or V lesions in breast imaging and underwent subsequent excision biopsy (confirmed seven invasive tumours and one DCIS) because of disagreement between sonographic and histological finding in LCNB specimen. Only three lesions classified as benign by means of breast imaging and LCNB came up to be malignant (two invasive cancers, one DCIS) at excision biopsy. None of the recorded parameters like lesion size, palpability or number of biopsies was significantly different in the group of malignant lesions not recognised by LCNB-based diagnosis.

Half of all masses (51.4%) were larger than 20 mm, 36.8% were between 10 and 20 mm, and 11.8% smaller than 10 mm.

Clinically significant complications (that required additional medical intervention as a consequence of the biopsy) occurred in one (0.1%) of all biopsied patients: an infection required surgical drainage and antibiotic treatment. Cases of minor interstitial haemorrhage, ecchymosis, or self-limiting inflammation were not considered to be significant complications.

## DISCUSSION

Large-core needle biopsy is considered the gold standard procedure for diagnosis of palpable and nonpalpable breast lesions, preferred because it allows a characterisation of benign and malignant lesions by histological examination (Parker *et al*, 1994; Philpotts *et al*, 2003; Pijnappel *et al*, 2004). In comparison, fine-needle-aspiration (FNA) which yields a cytological specimen shows a higher percentage of inconclusive results up to 29% (Klijanienko *et al*, 1998). This is a known disadvantage of this method. A multi-institutional clinical trial to evaluate different strategies of image-guided breast intervention showed a percentage of insufficient samples between 22 and 46% (Pijnappel *et al*, 2004). Furthermore, the success of FNA is operator dependent and relies on pathologists with a profound knowledge in cytopathology. This is in accordance with other studies (Pisano *et al*, 1998; Farshid and Rush, 2003). However, the major problem of FNA is the fact that it is not capable of distinguishing between DCIS and infiltrating carcinoma, mostly resulting in a DCIS overestimation rate (Pijnappel *et al*, 2004). Since there is a difference in treatment of DCIS and infiltrating carcinoma with respect to axillary lymphonodectomy, this is a serious clinical problem.

US-guided LCNB in a large number of patients has first been described and evaluated in a multi-institutional study by Parker *et al* (1994), who used an automated core biopsy device fitted with 14-gauge needles. Needle guidance was accomplished by means of either a stereotactic device or ultrasound imaging. Subtracting those that have been biopsied stereotactically by mammographic guidance, 1408 breast lesions were biopsied in 20 different institutions under ultrasound control. The false-negative rate of 4% was similar to that of the open surgical biopsy, for which 0.2–20% was reported (Rissanen *et al*, 1994; Jackman and Marzoni, 1997; Pijnappel *et al*, 2004). However, previously published studies lack consistent data indicating how many biopsies are necessary to achieve a reliable histological finding. There is only one study on a small number of 73 breast lesions that pays attention to that question (Fishman *et al*, 2003). This investigation consisted of 14 malignant and 59 benign lesions. They conclude that at least four cores have to be obtained to get a reliable diagnosis.

We demonstrate the results from 962 LCNB and show that three core needle samples, which were validated by 3D-US to confirm the correct placement of the biopsy needle, are sufficient for a reliable histological classification of breast tumours. We use a multi-planar display to define the precise post-firing position of the LCNB track, because needle visualisation by 2D within a lesion may be somewhat more subjective and be influenced by partial-volume effects towards the periphery of the lesion that might reduce the yield for small lesions. Furthermore, longitudinal visualisation of the complete needle track is mostly not carried out in the daily routine. The additional orthogonal plane, however, yields the multi-planar visualisation of the 3D ultrasound, proves the proper placement of the LCNB needle, and can be documented. This information cannot easily be replaced by only turning the probe by 90° (Weismann *et al*, 2000; Smith DN *et al*, 2001; Smith WL *et al*, 2001).

The false-negative rate of 3.9% (24 out of 615) and the number of insufficient samples of 0.4% in our study (four cases: one patient refused, the other three underwent excision biopsy; those cases have been excluded from statistical analysis) confirmed the results of other investigations (Parker *et al*, 1994; Lee *et al*, 1999; Pijnappel *et al*, 2004). Besides lesions that could undoubtedly be classified as benign or malignant, there is a fraction (termed histological underestimates) of histological findings allowing only an incomplete characterisation of the pathology. In this study, the LNCB-based diagnoses of the 11 DCIS (58%) underestimates for which subsequent analysis of the surgical specimen revealed invasiveness were higher than in other studies (Fishman *et al*, 2003; Philpotts *et al*, 2003; Pijnappel *et al*, 2004).

Obviously, there is a certain small risk of misdiagnosing a DCIS lesion to be an ADH, or malignant lesions to consist only of ADH or LCIS (two in our study) on the basis of small samples, which indicates the need for an open surgical approach in ADH and DCIS-classified cases. This is in accordance with the recommendation of consequent surgical intervention to remove all lesions of these types (Yeh *et al*, 2003). Invention of such general treatment strategy to avoid underestimation of some lesions would have reduced the false-negative rate in our study further from 3.9% (24 out of 615) to 1.8% (11 out of 615); the sensitivity would have increased to 98.2%, with a constant specificity of 100%. The high sensitivity achieved in this study with 3D-US-guided LCNB also reflects that patients with microcalcifications, for which this technique may not be feasible, were not included and always biopsied under mammographic guidance.

Nine invasive cancers were found by open surgical biopsy despite benign diagnosis after core needle biopsy. Since that histological finding was not in concordance with a suspicious sonographic appearance, eight of these patients underwent open biopsy that revealed seven invasive cancers and one DCIS. Subtracting these cases in which combined information of both breast imaging and the LCNB eventually resulted in the identification of malignant lesions, only three remaining cases of true core misses (two invasive cancers, one DCIS) would have led to false-negative diagnosis and subsequent suboptimal treatment of the patients. Fortunately, one of these lesions was removed at the patient's request and two as a consequence of complementary diagnosis.

Reasons to obtain a sufficient, but not excessive, number of biopsy samples include the minimisation of procedure time, patient discomfort, and breast trauma. Compared to surgery, it is less invasive and causes minimal to no scarring. This is an important issue in view of problems to assess scar tissue accurately in breast imaging. In our study, five of 11 (45%) core misses were observed in scars from former excision biopsies (more than 1 year before) where either sonographic assessment of the lesion or visualisation of the needle was impaired. This is supported by our observation that five of the 11 core biopsy misses later diagnosed to be invasive carcinomas had been expected to be scar tissue from former surgeries.

Nevertheless, LCNB is considered to obviate unnecessary surgery for many women with benign breast lesions by warranting a reliable histological finding, since 70–80% of breast lesions referred for biopsy in a nonselected collective of patients are benign (Rissanen *et al*, 1994; Crowe *et al*, 2002).

In conclusion, we demonstrate that at least three core needle biopsies performed under 3D-US validation are sufficient to obtain a reliable histological diagnosis of breast lesions. This technique could, therefore, replace surgical biopsy as the standard technique to remove tissue for initial histological assessment of breast lesions.

## Figures and Tables

**Table 1:** Characteristics of single cases of core needle biopsy misses (complete disagreement)

	**Sonographic appearance**	**Palpability**	**Histological finding at core needle specimen**	**Histological finding at surgical specimen**	**Lesion diameter**	**Number of cores**
1	Suspect	No	Scar-tissue	Infiltrating ductal carcinoma	15	2
2	Suspect	No	Scar-tissue	Infiltrating papillary carcinoma	10	2
3	Suspect	No	Inflammation	Infiltrating ductal carcinoma	18	2
4	***Benign***	***Yes***	***Fibrocystic change***	***Ductal carcinoma*** *in situ*	***ND*^a^**:	***3***
5	***Benign***	***Yes***	***Inflammation***	***Infiltrating ductal carcinoma***	***32***	***3***
6	***Benign***	***Yes***	***Scar tissue***	***Infiltrating carcinoma* b**	***55***	***3***
7	Suspect	Yes	Fat tissue	Infiltrating mucinous carcinoma	28	5
8	Suspect	Yes	Fat tissue	Infiltrating ductal carcinoma	28	2
9	Suspect	No	Scar tissue	Infiltrating ductal carcinoma	7	2
10	Suspect	Yes	Scar tissue	Infiltrating ductal carcinoma	25	3
11	Suspect	No	Fibrocystic change	Ductal carcinoma *in situ*	2	2

a ND: no data.

b Not further classified.

The bold characters are used to emphasise groups of cases containing true core needle biopsy misses.
